# Diet Quality and Weight Gain Among Women in the Aboriginal Family Birthing Program in South Australia

**DOI:** 10.3390/nu18183102

**Published:** 2026-09-21

**Authors:** Casey Cameron, Mohammad Radwanur Talukder, Jessica Reid, Philippa Middleton, Cathy Leane, Kim Morey, Fiona Kathleen Mensah, Alice Rumbold, Maria Makrides, Megan Warin, Deanna Stuart-Butler, Karen Hawke, Amanda Collins-Clinch, Janiene Deverix, Angela Cavallaro, Karen Glover

**Affiliations:** 1 Women and Kids Theme, South Australian Health and Medical Research Institute (SAHMRI), Adelaide, SA 5000, Australia; casey.cameron@sahmri.com (C.C.); radwan.talukder@sahmri.com (M.R.T.); jessica.reid@kokatha.com.au (J.R.); philippa.middleton@sahmri.com (P.M.); fiona.mensah@mcri.edu.au (F.K.M.); alice.rumbold@sahmri.com (A.R.); maria.makrides@sahmri.com (M.M.); karen.hawke@sahmri.com (K.H.); 2 Global Health and Migration Unit, Department of Women’s and Children’s Health, Uppsala University, 75310 Uppsala, Sweden; 3 College of Health, Adelaide University, Adelaide, SA 5000, Australia; 4 Women’s and Children’s Health Network, SA Health, Adelaide, SA 5006, Australiaangela.cavallaro@sa.gov.au (A.C.); 5 Wardliparingga Aboriginal Health Research Unit, South Australian Health and Medical Research Institute, Adelaide, SA 5000, Australia; kim.morey@sahmri.com; 6 Intergenerational Health, Murdoch Children’s Research Institute, Parkville, VIC 3052, Australia; 7 Department of Paediatrics, The University of Melbourne, Melbourne, VIC 3052, Australia; 8 College of Education, Behavioural and Social Sciences, Adelaide University, Adelaide, SA 5005, Australia; megan.warin@adelaide.edu.au; 9 Faculty of Medicine, The University of Queensland, Brisbane, QLD 4101, Australia; deanna.stuart-butler@mater.uq.edu.au; 10 College of Medicine and Public Health, Flinders University, Adelaide, SA 5042, Australia; 11 Aboriginal Health Council of South Australia, Adelaide, SA 5000, Australia; amanda.collins-clinch@ahcwa.org; 12 Child and Family Health Services, SA Health, Adelaide, SA 5000, Australia; janiene.deverix@sa.gov.au

**Keywords:** Aboriginal Australian, pregnant women, diet quality, South Australia, nutrition, healthy eating, ARFS

## Abstract

Background: This study assessed diet quality and the association between engagement with nutrition support bundles and weight gain among pregnant women having Aboriginal and Torres Strait Islander babies who participated in the Aboriginal Family and Baby Bundles implementation project at two South Australian maternity sites. Methods: Cross-sectional analysis of dietary data was performed using the Australian Recommended Food Score (ARFS) at enrolment, and 24-h food group servings were compared between enrolment and late pregnancy visits. A composite variable for engagement with nutrition support bundles (low vs. high engagement) used initiation, intensity, and duration of engagement criteria. Separate regression models were applied to examine associations between ARFS, adherence to 24-h food serve recommendations, and maternal characteristics at enrolment. An exploratory univariable association between engagement status and gestational weight gain was examined. Results: The median ARFS score at enrolment was 39 (maximum of 62 out of a total of 73). Women aged above 25 years showed significantly higher ARFS compared with those aged 25 years and below (adjusted β coefficient 3.28, 95% CI 0.38, 6.19, *p* = 0.027). The number of participants meeting the recommended intake of daily fruit serves increased significantly at the late pregnancy visit (McNemar test *p* value = 0.004). Across pregnancy, consumption of energy-dense soft drinks or snack foods was common (60–68%). Only 15.4% (8/52) of participants showed adequate weight gain, while 26.9% (14/52) showed inadequate weight gain, and 57.7% (30/52) showed excessive weight gain, as per the recommendations for total gestational weight gain. In the high nutrition support engagement group, a higher proportion of adequate weight gain (24.0% vs. 7.4%) and a lower proportion of excessive weight gain were observed (28.0% vs. 85.2%) (*p* ≤ 0.001) compared with the low engagement group. Conclusions: This study identified sub-optimal diet quality, including high intake of energy-dense food throughout pregnancy and high proportions of inadequate and excessive weight gain among women having Aboriginal babies in South Australia. Further research is needed to determine the most appropriate ways to support healthy eating in pregnancy among women in South Australia.

## 1. Introduction

Optimum diet quality and nutrition before and during pregnancy play important roles in achieving healthy pregnancy and birth outcomes, improving perinatal survival, as well as providing a foundation for better long-term health of the mother and baby [1,2]. Sub-optimal maternal nutritional status may cause abnormal fetal growth patterns, including low birthweight (<2500 g), small for gestational age or growth restriction, large for gestational age, and macrosomia, and consequently increase the risk of developing childhood and adult chronic diseases [1]. In contrast, quality diets containing essential nutrients such as folic acid, vitamin D, iron, calcium, and omega-3 fatty acids during pregnancy can reduce the risk of adverse pregnancy outcomes, including gestational diabetes mellitus, preterm birth, obesity-related complications, gestational hypertension, and preeclampsia [3,4].

Healthy eating during pregnancy remains an important area for improvement among both non-Indigenous and Aboriginal and Torres Strait Islander women in Australia, as most pregnant women report not meeting recommended dietary intake guidelines [5,6,7]. The Australian Dietary Guidelines (ADG) provide evidence-based recommendations about the amount and types of foods and drinks that are needed for health and wellbeing across the life course, including for pregnant women [8]. To assess adherence to national dietary guidelines (e.g., ADG), different diet quality assessment techniques have been applied [9]. The Healthy Eating Quiz (HEQ) is a food-based diet quality index modeled on the Australian Recommended Food Score (ARFS) and has demonstrated validity as an estimate of usual nutrient intakes in toddlers, children, adolescents, and adults [10]. The HEQ captures intake of a variety of foods within food groups recommended in the ADG, and higher scores reflect greater consistency of dietary patterns with the guidelines [10,11].

Longitudinal research in Western Australia and New South Wales (NSW), Australia, reported sub-optimal ARFS (ranging from 34 to 38 across pregnancy, with a maximum of 73) among women during pregnancy [5,6]. Recent research across eight remote communities in Central Australia and Far North Queensland reported that daily servings of most of the core food groups (vegetables, fruit, grains, and dairy) among Aboriginal pregnant women were below the ADG-recommended quintiles [12]. More than 90% of pregnant Aboriginal and Torres Strait Islander Australians (*n* = 58) from the Gomeroi gaaynggal cohort study in NSW reported a higher intake of energy-dense, nutrient-poor foods than recommended, while only one-third of the participants reported the recommended intake of dairy, protein and vegetables as per the Australian standards [7]. Sub-optimal intakes of iron, calcium, and folate were also reported [13]. However, little is known about the diet quality of pregnant women in South Australia (SA), including among women carrying an Aboriginal and Torres Strait Islander baby.

Moreover, evidence suggests that maintaining a healthy diet and good nutrition during pregnancy can support adequate gestational weight gain (GWG) [14]. Adequate GWG plays an important role in achieving healthy maternal and birth outcomes as well as preventing long-term health risks [15]. While inadequate GWG is associated with an increased risk of preterm birth, low birthweight, and infants born small-for-gestational age, excessive GWG is associated with elevated risks of gestational diabetes, gestational hypertension, infants born large-for-gestational age or with macrosomia, and cesarean deliveries [15,16]. In a recent systematic review and meta-analysis of 40 studies across six WHO world regions, 68% of pregnant women had weight gain that was above or below the recommended [16]. Very few studies have reported GWG in Australia, including among Aboriginal and Torres Strait Islander women.

For Aboriginal and Torres Strait Islander Australians, following colonization, the transition from hunter–gatherer lifestyle practices led to reduced consumption of traditional foods rich in protein, fiber, and micronutrients, and consequently increased dependence on Western dietary practices (high in sugar, starch, fat, and salt) [17]. This shift has been associated with socio-economic disadvantages and poor diet-related health outcomes [17]. Aboriginal and Torres Strait Islander Australians continue to experience widespread economic and social disadvantages and poorer health outcomes, such as earlier onset and higher rates of chronic conditions and lower life expectancy than non-Indigenous people [18]. At the community consultations, Aboriginal communities in SA identified improved nutrition for pregnant women and babies as a high priority. However, little is known about the diet quality and dietary intake of pregnant Aboriginal and Torres Strait Islander and non-Indigenous women in SA. Although one study reported sub-optimal diet quality of pregnant non-Indigenous women in SA, it did not examine factors associated with adherence to recommended diet quality among South Australian women [19]. A clear understanding of dietary patterns and their determinants will therefore help promote effective and equitable nutrition strategies and health service planning for pregnant women.

Therefore, the objectives of the current study, among women carrying Aboriginal and Torres Strait Islander babies who participated in the Aboriginal Family and Baby Bundles (ABFABB) implementation research project in South Australia, were to:

1. Describe maternal diet quality and examine the sociodemographic characteristics associated with diet quality during early pregnancy;
2. Describe and assess changes in dietary intake from early to late pregnancy, compare intake at each time point with national guideline recommendations, and examine the sociodemographic characteristics associated with dietary intake during early pregnancy;
3. Conduct, in a small subgroup, an exploratory analysis of gestational weight gain and the association between engagement with nutrition support bundles and gestational weight gain.

## 2. Methods

### 2.1. Study Design and Settings

The Aboriginal Family and Baby Bundles (ABFABB) implementation research project was embedded in the well-established, coordinated, culturally safe, and respectful Ngangkita Ngartu (Aboriginal Family Birthing Program (AFBP), Women’s and Children’s Hospital (WCH)) in South Australia [20]. At the community consultations before commencement of the study, Aboriginal communities in SA had identified improved nutrition as a high priority, particularly for pregnant women, babies, and young families. Through extensive community consultation, ‘bundles’ were designed to build social relationships with women and families, to improve nutritional literacy, to help women and families access nutritious foods, to be physically active, to breastfeed if possible, and to follow Australian guidelines for baby and infant feeding. Through our partnership with a South Australian food delivery service, we provided women and their families with conveniently home-delivered fresh seasonal fruit, vegetables, and protein sources. Essentials Gift cards or Foodbank vouchers were also given to some participants to assist with buying groceries. Participants were provided with a range of practical support items within different bundles, such as kitchen items, healthy oils (olives, avocado, and macadamia), resources, and recipes for healthy eating. Following discharge from the Ngangkita Ngartu program of care (approximately 6 weeks postpartum), care was transferred to Child and Family Health Services (CaFHS) when possible.

The study was approved by the Human Research Ethics Committee at the Women’s and Children’s Health Network (HREC/17/WCHN/176; SSA/18/WCHN/029), and the Aboriginal Health Research Ethics Committee managed by the Aboriginal Health Council of SA (# 04-17-738).

The bundles were intended to be provided at specific times to suit milestones, e.g., soon after pregnancy confirmation, later in pregnancy, early post-birth, and up until the child was 12 months of age, and 4 bundles were planned during pregnancy from <16 weeks to 32 weeks. However, during COVID-19, our access to the antenatal clinic was heavily restricted, limiting our ability to continue direct face-to-face engagement with participants and recruit new participants and resulting in changes to the delivery of bundles. With these changes in delivery of the bundles, we also monitored participants’ engagement with the nutrition support bundles, including how early in pregnancy they joined the nutrition support, how many visits and contacts they had with the study team, and the duration of involvement in the nutrition support.

In the current study, we conducted a series of analyses within the ABFABB implementation research project, primarily including a cross-sectional analysis of dietary data collected at 16–24 weeks and 30–32 weeks of pregnancy, and we compared within-person changes in dietary intake between the two time points. ABFABB aimed to achieve healthy weight gain for mothers during pregnancy and up to 12 months post-partum. This objective was not based on an a priori hypothesis that required predefined historical cohort data for comparison of gestational weight gain. Given the restrictions on the intended study implementation, an exploratory analysis was carried out to examine the association between engagement with nutrition bundles and gestational weight gain in a small subgroup of participants with weight data available at two time points.

### 2.2. Recruitment and Data Collection

**Figure 1:**
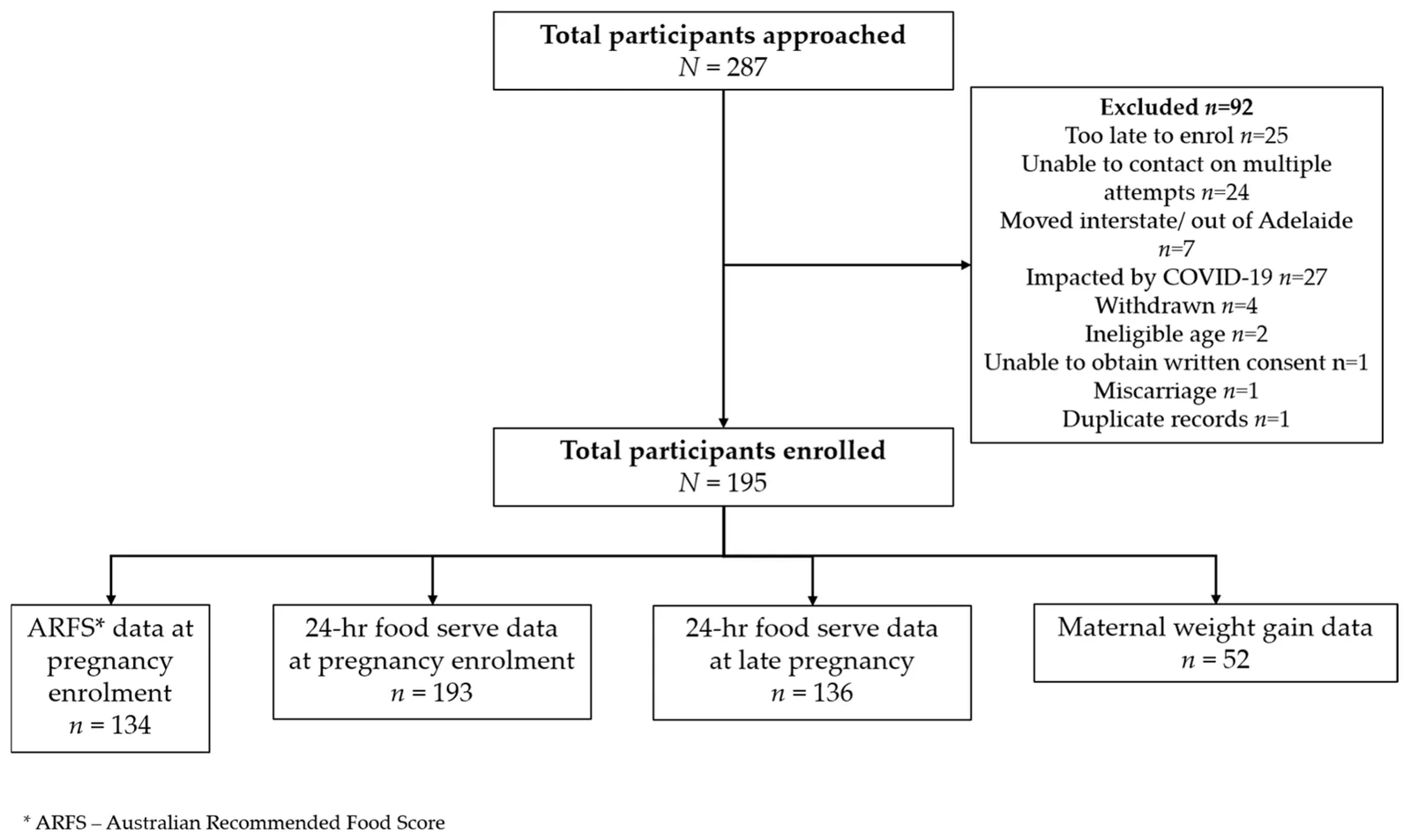
Number of participants with completed data available for different outcomes in this study.

Participants were recruited between April 2018 and June 2021. Aboriginal and non-Indigenous women giving birth to an Aboriginal and Torres Strait Islander baby, aged 16 or older, living within the Adelaide metropolitan region, and entering the Ngangkita Ngartu program were asked if they would like to participate in an additional flexible nutritional support program embedded in the Ngangkita Ngartu and continuing into early childhood services provided by CaFHS. No formal sample size calculation was done as the study applied descriptive and regression analyses to examine diet quality and diet intake and undertook an exploratory analysis of gestational weight gain based on the available data. Of the 287 participants who consented to be contacted, 92 were excluded during enrolment (Figure 1). Of the 92 participants excluded, 25 participants were too late to enroll, 24 participants were unable to be contacted, including due to a change in phone number or address, 7 participants moved interstate or out of Adelaide, 4 participants had withdrawn, 27 participants’ appointments had been impacted by COVID-19, we were unable to obtain written consent from 1 participant, 1 participant had a miscarriage, 2 participants were ineligible because they were below the eligible age, and 1 participant had duplicate records (Figure 1). Aboriginal research assistants enrolled participants and conducted the interviews at varying time points during pregnancy (<16 weeks, 18–20 weeks, 24 weeks, 30–32 weeks). The first time point data collection took place at a median of 23 weeks (range 10–35 weeks), and the second time point data collection took place at a median of 33 weeks (range 28–39 weeks). During COVID-19, restricted in-person contact at the antenatal clinic impacted data collection and the provision of bundles. For families already enrolled, phone calls were made instead of face-to-face visits for follow-up, and for new enrolments, all interactions with participants were by phone for completion of questionnaires.

#### Maternal Characteristics

Maternal weight was measured during early to mid-pregnancy during antenatal visits using the A&D UC-321 scale (A&D, Tokyo, Japan). However, access to routinely collected antenatal data, including height, required retrospective consent from participants and was therefore available for 94 participants.

Pre-pregnancy body mass index (BMI) was estimated as a weight-to-height ratio (weight in kilogram/height in meter^2^), applying a formula described elsewhere [21,22]. For women up to 20 weeks of gestation, 3 kg was subtracted from the current weight. For women who were over 20 weeks of gestation, 3 kg plus 0.5 kg/week for each week above 20 weeks was subtracted from their current weight. (Formula to obtain pre-pregnancy weight: weight in pregnancy − 3 kg if gestation week ≤ 20 weeks and pre-pregnancy weight = weight in pregnancy − (3 kg + (0.5 kg × (gestation week − 20))) if gestation week > 20 weeks). BMI was categorized as underweight (BMI < 18.5 kg/m^2^), normal weight (BMI 18.5–24.9 kg/m^2^), overweight (BMI 25.0–29.9 kg/m^2^), or obese (BMI ≥ 30.0 kg/m^2^). A sensitivity analysis was also performed to compare the BMI categories between pre-pregnancy BMI obtained from estimated weight and actual pregnancy BMI calculated at ≤16 weeks using the IOM-recommended weight gain range for the first trimester (0.5–2 kg) (*n* = 16) (Supplementary Table S1). The analysis showed consistent BMI classification using estimated pre-pregnancy BMI categories, except for 1 woman who was categorized as underweight when applying the IOM-recommended weight gain range [23]. Participants’ residential postcodes during enrolment were categorized into disadvantaged areas (postcodes within quintiles 1–3) and advantaged areas (postcodes within quintiles 4–5) based on the Index of Relative Socio-economic Advantage and Disadvantage (IRSAD) classification [24].

### 2.3. Outcome Measures

The primary outcomes of this paper were diet quality in pregnant women, assessed using the Australian HEQ at enrolment, and dietary intake, assessed using a 24-h recall questionnaire at enrolment and the late-pregnancy visit.

We also assessed incremental weight gain between the 2nd and 3rd trimesters of pregnancy, measured by calculating the weight difference divided by the number of weeks between the two occasions [23]. Participants’ total gestational weight gain (GWG) was also calculated by subtracting the estimated pre-pregnancy weight from the final weight in pregnancy and was categorized into inadequate, adequate, and excessive weight gain based on the IOM guideline range values for each pre-pregnancy BMI category [23].

#### 2.3.1. Healthy Eating Quiz and 24-h Dietary Intake

The HEQ [10,11], a 70-item food frequency questionnaire based on the ADG recommendations, was assessed at baseline in person when women were enrolled in the study. The HEQ assessed the number of different food groups eaten during a week. Participants were also asked to complete the 24-h diet questionnaire at every visit to recall consumption of serves of fruit, vegetables, beverages (including soft drink, juice, or cordial), and snack foods (hot chips, potato chips, biscuits, donuts, cakes, etc.) from the previous day using a standard questionnaire previously used in the Aboriginal population [25] and reviewed for appropriateness by the Aboriginal Governance Group of the study. Measuring cups and spoons were used as visual prompts to help participants with the serving size. For engagement during the COVID-19 period, serving size was determined by asking the same prompts over the phone. Daily serves of vegetables, fruits, and energy-dense foods (soft drinks, cordial or juice, or snack foods) were assessed as per the ADG recommendations [8]. The recommended daily servings of vegetables are ≥5 serves and fruit are ≥2 serves. The guideline recommends only occasional consumption of energy-dense foods such as soft drinks, cordial or juice, or snack foods, and in small amounts, to maintain a healthy weight in pregnancy [8]. Therefore, consumption of soft drinks and snack foods was presented as the percentage of participants consuming less than 1 serve on the previous day. Maternal factors associated with adherence to recommendations for 24-h food serves for vegetables and fruits or less than 1 serve on the previous day for soft drinks and snack foods at enrolment were also assessed.

#### 2.3.2. Australian Recommended Food Score

The Australian Recommended Food Score (ARFS) was derived from the HEQ responses [10]. The ARFS includes eight food group subscales: vegetables (0–21), fruit (0–12), protein foods (meat and fish) (0–7), meat alternatives (vegetarian sources of protein) (0–6), grain (0–13), dairy (0–11), water (0–1) and condiments (0–2), which in total give possible scores ranging from 0 to 73. Briefly, 1 point is awarded for each food item consumed according to frequency of consumption, with some healthy foods receiving bonus points (e.g., 2 points for ≥5 nights per week with meals containing vegetables, 2 points if the usual bread choice is multigrain or wholemeal, and 2 points if the usual type of milk is reduced-fat milk, or skim milk, or soy milk). Discretionary foods do not contribute to the calculation of the diet quality score [10]. The ARFS was calculated by adding the points for each item. A higher score indicates greater alignment with the ADG, and therefore higher diet quality. The total ARFS was classified into needs work or getting there (38 and below) and excellent or outstanding (39 and above) [5,6].

### 2.4. Engagement with Nutrition Support Bundles

We created a composite variable for engagement with nutrition support bundles that is easily understandable and replicable to assess the relationship with incremental weight gain, following the methods described elsewhere [26]. Briefly, the nutrition support engagement status variable comprised composites of: (1) engagement initiation (when in pregnancy the participants started receiving the bundles) (median 23 weeks); (2) engagement intensity (number of nutrition bundles the participants received) (received 2 or more bundles); and (3) engagement duration (number of weeks the participants were involved in the nutrition support) (median 12 weeks). First, we created two categories for engagement initiation and engagement duration, dividing at the median: participants who had started earlier in their pregnancy (≤23 weeks) vs. later (>23 weeks) and participants who stayed in the nutrition support for a longer period (>12 weeks) vs. a shorter period (≤12 weeks). Engagement intensity was defined by whether the participants received 2 or more bundles before birth (yes or no). Participants who scored high on at least 2 of the above 3 variables (initiation, intensity, and duration) were classified as having high nutrition support engagement status.

### 2.5. Statistical Analysis

Descriptive statistics (mean, standard deviation (SD), and frequency) as appropriate for categorical and continuous variables, were applied to describe maternal and demographic characteristics of participants. The measures of diet quality and diet intake included ARFS and 24-h serves for each food item and were presented as the median (interquartile range (IQR)) for consistency. The proportion of participants who consumed ADG-recommended food serves on the previous day at enrolment and at a late pregnancy visit was calculated. McNemar’s test was performed for paired comparisons of whether ADG-recommended food serves were met between early-pregnancy and late-pregnancy visits. Univariable and multivariable linear regression models were applied to assess the association between ARFS (dependent variable) and maternal characteristics (age, Aboriginal status, gestational age at enrolment (<23 and ≥23 weeks), and IRSAD classifications). The original model was also controlled for COVID-19 (i.e., enrolment before 2020 vs. during or after 2020) to control for any effect of COVID-19 on the ARFS. Adherence to 24-h food serve recommendations for vegetables and fruits, or less than 1 serve on the previous day for soft drinks and snack foods, was examined by applying the above maternal characteristics in univariable logistic regression models. Because of the low number of events in some outcomes (e.g., 24-h vegetable serves) and potential sparse data bias, Firth-penalized logistic regression was applied for multivariable models [27]. Covariate selection in the regression models was informed by prior literature [5] and based on the availability of data in the study, with models adjusted for age group, Aboriginal status, gestational week at enrolment, pre-pregnancy BMI (only in the sensitivity analysis model for ARFS) and IRSAD classifications. Linearity, homoscedasticity, and multicollinearity were assessed for linear regression model assumptions by applying residual plots and variance inflation factor (VIF) diagnostics. Diagnostic plots, supporting the reliability of regression estimates, demonstrated approximately linear relationships of residuals with fitted values and constant variance, and an approximately normal distribution of residuals; all VIF values (mean VIF = 1.02; individual VIFs < 1.1) indicated low multicollinearity across models. The categorical variable of total gestational weight gain was compared using Fisher’s exact test, while the mean of incremental weight gain between the 2nd and 3rd trimesters was compared using an independent *t*-test between high and low nutrition support bundle engagement status. No adjusted analysis related to gestational weight gain was conducted due to the small sample size (*n* = 52). All data manipulation and statistical analyses were performed using STATA, version 19 (Stata Corp., College Station, TX, USA).

## 3. Results

Of the 195 women enrolled in the study, 134 (68.7%) had diet quality data from the HEQ, 193 (99.0%) had 24-h food serve data at pregnancy enrolment, and 136 (69.7%) had 24-h food serve data in late pregnancy available for the analyses (Figure 1).

### 3.1. Characteristics of Study Participants

Table 1 summarizes the demographic characteristics of participants. The mean (SD) gestational age at the time of enrolment was 23.0 (5.0) weeks (*n* = 187). The mean (SD) age of participants was 26.4 (5.7) years, and 44.5% of participants were ≤25 years of age. Fifty-two percent of participants were overweight or obese based on estimated pre-pregnancy BMI (*n* = 94). Fifty-six percent of participants resided in postcodes classified as disadvantaged areas by the IRSAD index. No significant difference in characteristics of participants was observed between participants with complete data and those with incomplete data for each outcome of interest (Supplementary Table S2).

### 3.2. Australian Recommended Food Score (ARFS) at Enrolment

Table 2 presents the summary results of total ARFS and diet quality scores of participants (*n* = 134) by different food groups. None of the participants achieved the maximum ARFS of 73, and the highest ARFS was 62 (median 39, IQR 35–45). The diet quality of 45.9% (*n* = 62) of participants was in the ‘getting there or needs work’ category (ARFS score 38 and below). The median scores of different food groups separately were low to moderate relative to the maximum possible score for each group (Table 2). Relative to the total points available for each food group, vegetables (13 out of 21 points) and grains (8 out of 13 points) scored most highly, followed by fruit (6 out of 12 points) and meat (4 out of 7 points). Dairy and meat alternatives scored less than half the total points available at pregnancy enrolment, with meat alternatives being the lowest (two out of six points) (Table 2).

### 3.3. Association Between ARFS and Maternal Characteristics at Enrolment

In the adjusted linear regression model, ARFS remained significantly higher among participants aged above 25 years compared with those aged 25 years and below (β 3.28, 95% CI 0.38, 6.19, *p* = 0.027) (Table 3). Including pre-pregnancy BMI in the adjusted model of a smaller sample (*n* = 70) also did not attenuate this association (β 5.49, 95% CI 1.72, 9.26, *p* = 0.005) (Supplementary Table S3). Including COVID-19 in the original model, we observed a significant decline in ARFS during the COVID-19 period (β −3.31, 95% CI −6.48, −0.15, *p* = 0.04) (Supplementary Table S4).

### 3.4. 24-h Food Servings

Table 4 presents median (IQR) servings and the percentage of participants who consumed the ADG-recommended number of daily servings for food groups on the previous day during their pregnancy visits. The recommended number of daily servings of vegetables, i.e., ≥five serves, and fruit, i.e., ≥two serves, was achieved by only 7.4% (*n* = 14) and 27.2% (*n* = 52) of the participants, respectively, at early to mid-pregnancy (Table 4). The proportions of participants with no or minimal consumption of each category of energy-dense foods in the previous day were low (Table 4). In other words, 60.6% of the pregnant women had ≥one serve of soft drinks, cordial, or juice, i.e., at least 250 mL of soft drinks, cordial, or juice, and 67.2% had snack foods (e.g., hot chips, potato chips, biscuits, donuts, cakes) on the previous day at early pregnancy.

During the late pregnancy visit, the proportion of participants consuming the recommended daily fruit serves was higher (41.8%) (Table 4). In contrast, the proportion of participants reporting recommended serves of vegetables also remained low in late pregnancy (6.0%, *n* = 8/134). Sixty-three percent of participants consumed one or more serves of soft drinks, cordial, or juice at the late pregnancy visit (Table 4), and the percentage of participants consuming one or more serves of snack foods was similar at the late pregnancy visit (Table 4). McNemar’s test for paired comparison between the two visits showed that the number of participants who had recommended fruit servings on the previous day increased significantly at the late pregnancy visit (*p* = 0.004) (Supplementary Table S5). None of the other food serves (vegetables, soft drinks, and snack foods) changed significantly between the two time points (Supplementary Table S5).

### 3.5. Maternal Characteristics Associated with Adherence to Recommendations for 24-h Food Serves at Enrolment

In the adjusted model, participants who enrolled later in pregnancy were less likely to have the recommended fruit serves (adjusted odds ratio aOR 0.40, 95% CI 0.21, 0.79, *p* = 0.008) compared with those enrolled earlier in pregnancy. Participants aged >25 years were more likely to have the recommended intake of vegetable serves on the previous day compared with younger participants (Table 5). Aboriginal and/or Torres Strait Islander participants were more likely to have <one serve of snack foods on the previous day (aOR 2.74, 95% CI 1.03, 7.29, *p* = 0.044) in early pregnancy compared with non-Indigenous participants (Table 5).

### 3.6. Gestational Weight Gain and Engagement with Nutrition Support Bundles

An exploratory analysis of gestational weight gain and engagement with nutrition support bundles was performed in a smaller number of participants (*n* = 52, 27.0% of total participants, excluding one woman with biologically implausible incremental weight gain of 4 kg/week between the second and third trimesters, for whom an estimated pre-pregnancy weight and third-trimester weight were available for analysis. Only 15.4% (8/52) of participants had adequate weight gain, 26.9% (14/52) had inadequate weight gain, and 57.8% (30/52) had excessive weight gain as per the IOM recommendations (Supplementary Table S6). By pre-pregnancy BMI category, almost equal proportions of participants were within the recommended GWG category among the underweight or normal weight group (16%, 4/25) and the overweight or obese group (15%, 4/27) (Supplementary Table S6). The proportion of participants with adequate weight gain was higher (24.0% vs. 7.4%), and the proportion with excessive weight gain was lower (28.0% vs. 85.2%) in the high nutrition support engagement group (*p* ≤ 0.001). However, the proportion of participants with inadequate weight gain remained higher in the high engagement group. Examining the association between total weight gain and each engagement component separately showed significantly higher proportions of adequate weight gain and lower proportions of excessive weight gain among participants with earlier engagement with the nutrition support bundles and longer engagement duration. However, proportions of weight gain categories did not significantly differ by participants’ exposure to the number of bundles (Supplementary Table S7).

Mean incremental weight gain between the second and third trimesters (*n* = 52) was 0.45 kg/week (SD 0.32). This included six women with negative weight change. All negative weight changes occurred among overweight or obese women (two participants had missing pre-pregnancy BMI data). Negative weight change was also equally distributed by engagement status. Excluding the women with negative weight change, mean incremental weight gain was 0.52 kg/week (SD 0.25) (*n* = 46).

Overall, incremental weight gain did not differ between high- and low-nutrition support engagement groups except among women with positive weight change (Table 6). For women with positive weight change and in the high engagement group (*n* = 26), the mean incremental weight gain was 0.44 kg/week (SD 0.23), while in the low engagement group (*n* = 20) the mean incremental weight gain was 0.59 kg/week (SD 0.25) (*p* = 0.036) (Table 6). Incremental weight gain among all participants did not differ for any of the engagement components separately (Supplementary Table S7).

## 4. Discussion

Among pregnant women carrying Aboriginal and Torres Strait Islander babies in South Australia who were participating in a nutrition support program, we found low intakes of core food groups and high intakes of energy-dense, less nutritious foods across pregnancy, indicating sub-optimal diet quality. Our ability to assess the effectiveness of the nutrition support bundle strategies was severely impacted by the COVID-19 pandemic, which limited the full-scale implementation of the nutrition strategies and direct face-to-face interactions to promote healthy eating. Aboriginal and Torres Strait Islander peoples generally prefer face-to-face interaction that supports care providers in establishing trust, respect, and ensuring cultural safety, and results in better engagement and understanding in health contexts [28]. It was also found that ARFS was significantly lower among pregnant women who enrolled during COVID-19, indicating a significant impact of the COVID-19 pandemic on overall diet quality. With 80% of study participants being Aboriginal and Torres Strait Islander, this may partly explain the limited impact of nutrition support packages observed in this study.

Our observation of sub-optimal diet quality, relative to national healthy eating guidelines, is consistent with previous research conducted among Aboriginal and non-Indigenous pregnant women in Australia [6,7,29]. The median ARFS of study participants was higher than that reported in the Gomeroi gaaynggal study [7] as well as in research among non-Indigenous women [29] but similar to that reported among non-Indigenous women in the BABY1000 study [6]. However, in our research, 46% of participants had an ARFS of 38 or below, which highlights the need for further and more intensive nutrition support initiatives addressing social, economic, cultural, and environmental aspects [30] of healthy food intake among Aboriginal and non-Indigenous women during pregnancy to ensure optimal growth of offspring and prevent adverse maternal health outcomes.

In our study, the number of participants meeting the recommended number of daily servings for core food groups (e.g., vegetables and fruits) was low. Overall, across the pregnancy period, the intake of vegetable and fruit serves relative to the ADG recommendation was particularly low. While this contrasts with the reported serves in the Gomeroi gaaynggal study in NSW [7] it is comparable with that of Aboriginal women in another Australian study [12]. In the Gomeroi gaaynggal study, 29.3% of Aboriginal pregnant women met recommended daily vegetable serves [7] compared with 5.9% in late pregnancy in this study. The low proportion of participants adhering to recommended daily serves for vegetables and the relatively higher proportion of participants adhering to guideline-recommended daily serves for fruits are also consistent with recent research predominantly with non-Indigenous Australian pregnant women [31].

In pregnancy, the Australian Dietary Guidelines recommend only occasional consumption and small amounts of energy-dense, nutrient-poor food items but do not provide any specific daily limit [8]. In this study, more than 60% of participants had more than one daily serve of soft drink, cordial, or juice. Similarly, 68% of participants had more than one serve of snack foods daily. This suggests that higher intake of non-core foods may shift the energy intake from nutrient-dense core foods in the study population in SA. A similar diet pattern, i.e., below recommended intake of fruits and vegetables and above minimal intake of energy-dense foods, was also observed among Indigenous communities in Australia and internationally [30,32]. In contrast, among pregnant non-Indigenous women, more than two-thirds of energy intake was contributed by core food groups [5,6]. However, we also observed that pregnant Aboriginal women were more likely to have minimal (less than one serve) snack food intake on the previous day in early pregnancy compared to pregnant non-Indigenous women, which may be due to day-to-day variability in intake but also warrants further exploration. Inadequate intake of nutrient-rich core foods with high consumption of energy-dense non-core foods may increase the risk of adverse effects on mother–infant cardiometabolic health [1]. Therefore, health and nutrition literacy messages need to adopt effective strategies targeting the non-core food groups. These results also warrant further exploration to comprehensively understand the social and cultural contexts of unhealthy food intake among pregnant women and to explore the ways to promote adequate intake of different food groups by pregnant women in South Australia as recommended.

Twenty-four-hour reporting of intake of core and non-essential food groups following exposure to a bundle of interventions between two visits showed mixed results. Compared with baseline, the proportion of participants consuming fruit serves as per the ADG recommendation improved in the subsequent pregnancy visit. However, the proportion consuming recommended vegetable servings reduced, while the proportion consuming more than one serve of soft drink, cordial, or juice increased slightly in the subsequent visit in late pregnancy. These results need to be interpreted cautiously in the context of women’s day-to-day variability in intake. It is also possible that aversions and cravings for a particular food item (e.g., vegetables, soft drinks, etc.) during pregnancy [33] could contribute to day-to-day variability of food intake, which may have resulted in differential intake of food groups. Despite the absence of a substantial effect of nutrition packages, our research highlights that Australian women having an Aboriginal baby during their pregnancy need additional support to improve their diet quality and health outcomes.

Research on weight gain in pregnancy among Aboriginal families has been very scant [34]. Only 15% of Aboriginal pregnant women in the Gomeroi gaaynggal study (in regional and remote towns in NSW) had adequate gestational weight gain, while 32% had inadequate weight gain and 54% had excessive weight gain [34], when compared with the 2009 IOM recommendations and based on pre-pregnancy BMI [23]. While the proportions of inadequate, adequate, and excessive weight gain in this research were found to be consistent with the Gomeroi gaaynggal study, the proportion of participants with adequate gain was lower (15%), and the proportion with excessive weight gain (58%) was higher than that in the Gomeroi gaaynggal study [34]. Unlike the Gomeroi gaaynggal study, which had the highest proportion of excessive weight gain in overweight participants (74%) but a relatively lower proportion in the normal weight category (48%) [34], the proportion of participants with excessive weight gain in this research was consistent (~60%) across all BMI categories. The proportion of adequate weight gain in this research was also lower (15%) than that reported among American Indian or Alaskan Native people (29%) [35] and non-Indigenous Australian women (38%) [36]. We observed a higher proportion of adequate weight gain and a lower proportion of excessive weight gain in the high engagement status group compared to the low engagement status group in the univariable analysis. When examining the association of total weight gain categories with each engagement component separately, earlier engagement with the nutrition support bundles and a longer duration of engagement, rather than exposure to a greater number of bundles, showed significant associations (Supplementary Table S7). However, the classification of total gestational weight gain and the association between engagement status and gestational weight gain need to be interpreted with caution as exploratory rather than as a direct intervention effect because of the small number of participants, reliance on estimated rather than actual pre-pregnancy BMI, and potential confounding factors including age, parity, pre-pregnancy BMI, smoking, and diabetes. Nevertheless, the findings indicate greater disparity in adequate gestational weight gain within and between Aboriginal and non-Aboriginal women nationally and internationally. Given the known adverse consequences of non-optimum weight gain on maternal and child health outcomes as well as long-term health risks [15], all this evidence combined also highlights that greater community and health service efforts are needed to tackle the persistent problem of non-optimum GWG in Aboriginal and non-Indigenous women.

In terms of incremental weight gain, a study of non-Indigenous culturally diverse Australian women (*n* = 2313) in mid-second trimester reported a rate of 0.41 kg/week among underweight, 0.38 kg/week among normal weight, 0.45 kg/week among overweight, and 0.46 kg/week among obese groups [37]. A lower gain rate between the second and third trimesters in overweight (*n* = 7, 0.33 kg/week) and obese (*n* = 12, 0.35 kg/week) participants, but a higher rate in healthy weight participants (*n* = 11, 0.55 kg/week) was observed in this research. However, this comparison needs to be interpreted with caution because of the low number of participants within each pre-pregnancy BMI category and methodological differences in calculating weight gain in the mid-second trimester [37] vs. between the second and third trimesters in this research. Given the challenges of raising awareness about gestational weight gain through health education [34] and managing this through diet and lifestyle interventions only, particularly among overweight and obese women [38], more research is also required to determine the type and intensity of interventions that are culturally acceptable to achieve optimal weight gain in women during pregnancy [34].

The findings from our study suggest that further investments are needed to optimize dietary intakes and nutrition outcomes among pregnant women in South Australia and beyond. Food intake and diet quality during pregnancy were found to be stable [6,19] highlighting that a life-course healthy approach supported by appropriate social, cultural, and environmental strategies before pregnancy is required for maintaining healthy dietary changes across and beyond pregnancy for Aboriginal and non-Indigenous women. Moreover, among overweight and obese women, interventions only focusing on diet and lifestyle changes in pregnancy have had limited impact on gestational weight gain and adverse pregnancy outcomes [38]. Therefore, focus should be given to interventions prior to pregnancy to optimize maternal health and weight, improve pregnancy and birth outcomes, and prevent long-term health risks associated with obesity [38]. Systematic reviews of studies on nutrition interventions recommend multifaceted approaches that improve individual knowledge and skills, as well as strategies that increase access to nutritious food and provide a healthy food environment supported by co-design and Aboriginal and Torres Strait Islander leadership to promote nutrition outcomes in Aboriginal and Torres Strait Islander communities [39,40].

Our research adds to the evidence that pregnant women having Aboriginal babies in South Australia require additional support to optimize their diet quality and nutrition. The project was informed by priorities identified by the community and led by Aboriginal researchers under an established Aboriginal governance framework. However, results of this research need to be interpreted in the context of several limitations. The implementation of the project was heavily impacted by COVID-19, limiting our ability to engage with and follow up with some women. Participants’ availability to consent retrospectively to access their routine antenatal data from medical records resulted in incomplete height data capture for some participants and may have introduced missing data and potential selection bias. Because of missing data resulting in a small sample size, we were unable to perform an adjusted analysis when examining the association between engagement status and gestational weight gain. Therefore, observed differences should not be viewed as a causal effect of the intervention; instead, they identify areas for further research that include a robust control group. Further, a small number of women experienced gestational weight loss. This may represent a distinct physiological or clinical phenomenon or potential measurement error, and we were unable to determine the causes of weight loss in these women, which should be explored in future research. We used a standard questionnaire administered by Aboriginal research staff. We only obtained previous day food intake data, which is subject to day-to-day variability. In the absence of a recommended daily serve size for non-core food (e.g., snack foods or soft drinks), we applied less than one serve and more than one serve as cut-offs, which could be a source of misclassification bias. The pre-pregnancy BMI was calculated based on a specific formula to obtain pre-pregnancy weight [22], which was not validated in Aboriginal or Australian populations and could be a source of error. We used a proxy measure for women’s socio-economic status by applying the IRSAD classification, which is subject to ecological fallacy, i.e., group-level characteristics may not represent individual-level status.

## 5. Conclusions

Dietary intakes and weight gain of pregnant women having Aboriginal and Torres Strait Islander babies in South Australia were found to be inadequate relative to current national recommendations for pregnancy. More investment is required to co-design and evaluate culturally acceptable approaches combining nutrition education and interventions addressing food access to improve diet and nutrition outcomes of pregnant women, as well as to give greater focus to interventions commencing prior to pregnancy. Further research will also be useful to identify ways to address high intake of non-core energy-dense food groups and non-optimum weight gain in pregnant women in South Australia.

## Figures and Tables

**Table 1:** Characteristics of study participants.

Age (Years) (*n* = 191)	Mean	sd
	26.4	5.7
Age group	*n*	%
≤25 years	85	44.5
>25 years	106	55.5
Aboriginal status of mother (*n* = 195)	*n*	%
Aboriginal and/or Torres Strait Islander	156	80.0
Non-Indigenous	39	20.0
Gestational age at enrolment (*n* = 187)	Mean	sd
	23.0	5.0
Gestational age at enrolment category	*n*	%
<23 weeks	88	47.6
≥23 weeks	99	52.4
Estimated Pre-pregnancy BMI (kg/height in m^2^) ^a^ (*n* = 94)	Mean	sd
	27.2	7.8
Pre-pregnancy BMI category	*n*	%
Underweight (<18.5 kg/height in m^2^)	10	10.6
Normal weight (18.5–24.99 kg/height in m^2^)	35	37.2
Overweight (25–29.99 kg/height in m^2^)	17	18.1
Obesity (≥30 kg/height in m^2^)	32	34.0
Postcodes IRSAD ^b^ classification (*n* = 193)	*n*	%
Advantaged area (quintiles 4–5)	84	43.5
Disadvantaged area (quintiles 1–3)	109	56.5

^a^ Pre-pregnancy BMI—calculated by subtracting 3 kg from the current weight for women up to 20 weeks of gestation and 3 kg plus 0.5 kg/week from their current weight for women who were over 20 weeks of gestation. BMI could not be calculated for 101 (52%) participants because they were not available for retrospective consent to access their routinely collected antenatal data, including height. ^b^ IRSAD—Index of Relative Socio-economic Advantage and Disadvantage.

**Table 2:** Australian Recommended Food Score (ARFS) among participants at enrolment.

ARFS ^a^ (Maximum Score)	Median	IQR ^b^
Total foods (73)	39	35–45
Vegetables (21)	13	11–15
Fruit (12)	6	4–8
Protein foods ^c^ (7)	4	3–4
Meat alternatives ^d^ (6)	2	1–3
Grains (13)	8	7–9
Dairy (11)	5	3–6
Water (1)	1	1–1
Condiments (2)	2	1–2

^a^ ARFS—Australian Recommended Food Score; ^b^ IQR—Interquartile range; ^c^ protein foods—meat and fish; ^d^ Meat alternatives—vegetarian sources of protein.

**Table 3:** Linear regression analysis of ARFS total score at enrolment by maternal characteristics.

	Unadjusted β-Coefficient	95% CI	*p* Value	Adjusted ^b^ β-Coefficient	95% CI	*p* Value
Age group						
≤25 years	Ref					
>25 years	3.22	0.42, 6.02	0.024	3.28	0.38, 6.19	0.027
Aboriginal status of mother						
Aboriginal and/or Torres Strait Islander	−1.49	−5.11, 2.13	0.417	−0.13	−3.76, 3.49	0.943
Non-Indigenous	Ref					
Gestational age at enrolment						
<23 weeks	Ref					
≥23 weeks	0.36	−2.60, 3.33	0.809	0.79	−2.12, 3.69	0.592
Postcodes IRSAD ^a^ classification						
Disadvantaged area	Ref					
Advantaged area	0.54	−2.42, 3.50	0.719	0.94	−1.99, 3.87	0.526

^a^ IRSAD—Index of Relative Socio-economic Advantage and Disadvantage; ^b^ Adjusted for age, Aboriginal status of mother, gestational age at enrolment, and IRSAD classification.

**Table 4:** 24-h food intake history of participants during the first pregnancy visit and late pregnancy visit.

	First Pregnancy Visit (Median 23 Weeks)			Late Pregnancy Visit (Median 33 Weeks)		
Food Group Servings	N	Median (IQR)	% Meeting Recommendations ^b^	N	Median (IQR)	% Meeting Recommendations ^b^
Vegetables (servings/day)	190	1 (0–2)	14 (7.4)	134	1 (0–2.5)	8 (6.0)
Fruits (servings/day)	191	1 (0–2)	52 (27.2)	134	1 (0–2)	56 (41.8)
			% less than 1 serve on the previous day			% less than 1 serve on the previous day
Soft drink ^c^ (servings/day)	193	2 (0–3)	76 (39.4)	136	2 (0–4)	50 (36.8)
Snacks ^ac^ (servings/day)	192	1 (0–2)	63 (32.8)	136	1 (0–2)	44 (32.3)

^a^ snack food (e.g., hot chips, potato chips, biscuits, donuts, cakes); ^b^ According to the ADG food group recommendations for pregnant women. ^c^ ADG recommends only occasional and small amounts of energy-dense, nutrient-poor foods. Soft drink or snack foods—represents women who had no or less than 1 serve of soft drink, cordial or juice, or less than 1 serve of snack foods.

**Table 5:** Penalized logistic regression analysis for adherence to 24-h food servings recommendations at enrolment by maternal characteristics.

	Vegetables		Fruits		Soft Drinks		Snack Foods	
	Odds Ratio (OR) (95% CI)	*p* Value	OR (95% CI)	*p* Value	OR (95% CI)	*p* Value	OR (95% CI)	*p* Value
Age group								
≤25 years	Ref		Ref		Ref ^c^		Ref ^c^	
>25 years	**5.10 (1.11, 23.46)**	**0.037**	0.99 (0.52, 1.90)	0.98	1.56 (0.86, 2.84)	0.14	1.30 (0.70, 2.41)	0.413
Adjusted OR ^a^	**4.18 (1.01, 17.19)**	**0.048**	0.95 (0.48, 1.86)	0.879	1.57 (0.85, 2.89)	0.149	1.37 (0.71, 2.63)	0.344
Aboriginal status of mother								
Non-Indigenous	Ref		Ref		Ref		Ref	
Aboriginal and/or Torres Strait Islander	3.46 (0.44, 27.31)	0.239	1.51 (0.64, 3.56)	0.352	0.51 (0.25, 1.04)	0.065	**2.53 (1.05, 6.12)**	**0.039**
Adjusted OR	7.04 (0.40, 122.89)	0.181	1.58 (0.64, 3.90)	0.323	0.52 (0.24, 1.10)	0.087	**2.74 (1.03, 7.29)**	**0.044**
Gestational age at enrolment								
<23 weeks	Ref		Ref		Ref		Ref	
≥23 weeks	0.77 (0.25, 2.39)	0.651	**0.40 (0.20, 0.78)**	**0.007**	1.08 (0.60, 1.96)	0.795	1.23 (0.66, 2.28)	0.52
Adjusted OR	0.75 (0.24, 2.31)	0.615	**0.40 (0.21, 0.79)**	**0.008**	1.16 (0.63, 2.12)	0.639	1.05 (0.55, 2.00)	0.886
Postcodes IRSAD ^b^ classification								
Disadvantaged area	Ref		Ref		Ref		Ref	
Advantaged area	0.33 (0.09, 1.22)	0.096	0.88 (0.46, 1.69)	0.709	1.35 (0.75, 2.43)	0.309	**0.47 (0.25, 0.90)**	**0.023**
Adjusted OR	0.41 (0.11, 1.45)	0.165	0.92 (0.47, 1.83)	0.819	1.26 (0.69, 2.32)	0.453	0.53 (0.27, 1.03)	0.061

^a^ Model for each outcome was adjusted for age, Aboriginal status, gestational week at enrolment and IRSAD classification. ^b^ IRSAD—Index of Relative Socio-economic Advantage and Disadvantage. ^c^ ADG recommends only occasional and in small amounts for energy-dense, nutrient-poor foods. Soft drink or snack foods—Compared women who had no or less than 1 serve of soft drink, cordial or juice, or less than 1 serve of snack foods vs. those who had ≥1 serve of soft drink, cordial or juice, or snack foods as the reference. Bold values indicate significant *p* values (<0.05).

**Table 6:** Summary statistics of total gestational weight gain and incremental weight gain of women by nutrition support engagement category.

	Low Engagement Status	High Engagement Status	*p* Value
Total Weight Gain Category			
Adequate	2 (7.4)	6 (24.0)	<0.001
Inadequate	2 (7.4)	12 (48.0)	
Excessive	23 (85.2)	7 (28.0)	
Incremental weight gain category			
All pregnant women (*n* = 52) [Mean (SD ^a^) kg/week]	0.52 (0.34)	0.36 (0.29)	0.087
Women with positive weight change [Mean (SD) kg/week] (*n* = 46)	0.59 (0.25)	0.44 (0.23)	0.036

^a^ SD—Standard deviation.

## Data Availability

The datasets presented in this article are related to Aboriginal and Torres Strait Islander Australians and, therefore, are not readily available because of ethical restrictions. Reasonable requests to access the datasets should be directed to the Corresponding Author, Karen Glover—karen.glover@sahmri.com.

## Supplementary Materials

The following supporting information can be downloaded at: https://www.mdpi.com/article/10.3390/nu18183102/s1. Supplementary Table S1: Sensitivity analysis for classification of BMI between estimated pre-pregnancy BMI and BMI at ≤16 weeks pregnancy with lower and upper range weight gain in trimester 1. Supplementary Table S2: Study participants’ characteristics by data completeness for each outcome analysis. Supplementary Table S3: Sensitivity analysis of linear regression of ARFS total score at enrolment by maternal characteristics including BMI in the model. Supplementary Table S4: Sensitivity analysis of linear regression of ARFS total score at enrolment by maternal characteristics including COVID-19 in the original model. Supplementary Table S5: Paired comparison of 24-h food serves between early and late pregnancy visits. Supplementary Table S6: Total gestational weight gain by pre-pregnancy BMI category. Supplementary Table S7: Sensitivity analysis of gestational weight gain by each engagement component.

## Abbreviations

The following abbreviations are used in this manuscript:

ABFABB	Aboriginal Family and Baby Bundles
ARFS	Australian Recommended Food Score
ADG	Australian Dietary Guideline
BMI	Body Mass Index
CaFHS	Child and Family Health Services
GWG	Gestational weight gain
HEQ	Healthy Eating Quiz
IOM	Institute of Medicine
IQR	Interquartile range
IRSAD	Index of Relative Socio-economic Advantage and Disadvantage
NSW	New South Wales
OR	Odds ratio
SA	South Australia
SD	Standard deviation
WCH	Women’s and Children’s Hospital
