# Supplementary material for: Diet Quality and Weight Gain Among Women in the Aboriginal Family Birthing Program in South Australia
Source: Nutrients. 2026 Sep 21;18(18):3102. doi: 10.3390/nu18183102 (PMC13610292; doi:10.3390/nu18183102)
Supplement: Supplementary file 1 [file nutrients-18-03102-s001.zip › nutrients-4514428-supplementary.pdf]

Supplementary Table S1. Sensitivity analysis for classification of BMI between estimated pre-pregnancy BMI and BMI at ≤16 weeks pregnancy with lower and upper range weight gain in trimester 1

	**Estimated pre-pregnancy BMI**		**≤16 weeks pregnancy BMI applying 0.5kg weight gain**		**≤16 weeks pregnancy BMI applying 2.0kg weight gain**	
Pre-pregnancy BMI category	*n* =16	%	*n* =16	%	*n* =16	%
Underweight (<18.5 kg/height in m^2)	1	6.2	0	0	0	0
Normal weight (18.5-24.99 kg/height in m^2)	6	37.5	7	43.7	7	43.7
Overweight (25- 29.99 kg/height in m^2)	0	0	0	0	0	0
Obesity (≥30 kg/height in m^2)	9	56.3	9	56.3	9	56.3

Supplementary Table S2. Study participants' characteristics by data completeness for each outcome analysis

	**Diet quality data completeness**					**Diet intake data completeness at late pregnancy**					**Weight data completeness**				
**Characteristics**	**Complete data**		**Incomplete data**		***p value***	**Complete data**		**Incomplete data**		***p value***	**Complete data**		**Incomplete data**		***p value***
Total	*n*=134		*n*=61			*n*=136		*n*=59			*n*=52		*n*=143		
Age (years) (*n*=191)	*n*=131		*n*=60			*n*=133		*n*=58			*n*=52		*n*=139		
	Mean	sd	Mean	sd		Mean	sd	Mean	sd		Mean	sd	Mean	sd	
	26.4	5.8	26.5	5.7	0.92	26.7	5.9	25.7	5.2	0.235	27.6	6.3	26.0	5.5	0.084
Age group	*n*	%	*n*	%		*n*	%	*n*	%		*n*	%	*n*	%	
≤25 years	60	70.6	25	29.4	0.594	60	70.6	25	29.4	0.797	21	24.7	64	75.3	0.484
>25 years	71	70.0	35	33.0		73	68.9	33	31.1		31	29.2	75	70.8	
Aboriginal status of mother (*n*=195)	*n*	%	*n*	%	*n*	%	*n*	%	*n*	%	*n*	%	*n*	%	*n*
Aboriginal and/or Torres Strait Islander	107	68.6	49	31.4	0.938	109	69.9	47	30.1	0.938	41	26.3	115	73.7	0.808
Non-Indigenous	27	69.2	12	30.8		27	69.2	12	30.2		11	28.2	28	71.8	
Gestation weeks at enrolment (*n*=187)	*n*=132		*n*=55			*n*=130		*n*=57			*n*=52		*n*=135		
	Mean	sd	Mean	sd		Mean	sd	Mean	sd		Mean	sd	Mean	sd	

Characteristics	**Diet quality data completeness**					**Diet intake data completeness at late pregnancy**					**Weight data completeness**				
	**Complete data**		**Incomplete data**		***p value***	**Complete data**		**Incomplete data**		***p value***	**Complete data**		**Incomplete data**		***p value***
	23.2	4.6	22.5	5.9	0.358	23.0	5.1	23.1	4.7	0.868	23.9	4.5	22.6	5.2	0.128
Gestation weeks at enrolment category	*n*	%	*n*	%	*n*	%	*n*	%	*n*	%	*n*	%	*n*	%	
<23 weeks	62	69.7	27	30.3	0.791	67	75.3	22	24.7	0.103	22	24.7	67	75.3	0.369
≥23 weeks	70	71.4	28	28.6		63	64.3	35	35.7		30	30.6	68	69.4	
Estimated Pre-pregnancy BMI (kg/height in m[2]) [a] (*n*=94)	*n*=71		*n*=23			*n*=73		*n*=21			*n*=33		*n*=61		
	Mean	sd	Mean	sd		Mean	sd	Mean	sd		Mean	sd	Mean	sd	
	27.2	7.9	27.2	7.4	0.998	27.1	7.8	27.5	7.8	0.833	28.1	8.2	26.7	7.6	0.401
Pre-pregnancy BMI category	*n*	%	*n*	%	*n*	%	*n*	%	*n*	%	*n*	%	*n*	%	*n*
Underweight (<18.5 kg/height in m[2])	8	80.0	2	20.0	0.731	8	80.0	2	20.0	0.527	3	30.0	7	70.0	0.878
Normal weight (18.5-24.99 kg/height in m[2])	28	80.0	7	20.0		29	82.9	6	17.1		11	31.4	24	68.6	

	Diet quality data completeness					Diet intake data completeness at late pregnancy					Weight data completeness				
**Characteristics**	**Complete data**		**Incomplete data**		***p* value**	**Complete data**		**Incomplete data**		***p* value**	**Complete data**		**Incomplete data**		***p* value**
Overweight (25-29.99 kg/height in m^2)	13	76.5	4	23.5		11	64.7	6	35.3		7	41.2	10	58.8	
Obesity (≥30 kg/height in m^2)	22	68.7	10	31.2		25	78.1	7	21.9		12	37.5	20	62.5	
Postcodes IRSAD [b] classification (n=193)	*n*=133		*n*=60			*n*=134		*n*=59			*n*=52		*n*=141		
Advantaged area (quintiles 4-5)	57	67.9	27	32.1	0.781	57	67.9	27	32.1	0.677	24	28.6	61	71.4	0.654
Disadvantaged area (quintiles 1-3)	76	69.7	33	30.3		77	70.6	32	29.4		28	25.7	80	74.3	

[a] Pre-pregnancy BMI – calculated by subtracting 3 kg from the current weight for women up to 20 weeks of gestation and 3 kg plus 0.5 kg/week from their weight after 20 weeks of gestation for women who were over 20 weeks of gestation. BMI cannot be calculated for 101 (52%) participants as they were not available for retrospective consent to access their routinely collected antenatal data, including height.

[b] IRSAD - Index of Relative Socio-economic Advantage and Disadvantage

Supplementary Table S3. Sensitivity analysis of linear regression of ARFS total score at enrolment by maternal characteristics including BMI in the model (n=70)

	**Unadjusted β-Coefficient**	**95% CI**	**p value**	**Adjusted [b] β-Coefficient**	**95% CI**	**p value**
Age group						
≤25 years	Ref					
>25 years	3.22	0.42, 6.02	0.024	5.49	1.72, 9.26	0.005
Aboriginal status of mother						
Aboriginal and/or Torres Strait Islander	-1.49	-5.11, 2.13	0.417	1.28	-3.12, 5.68	0.562
Non-Indigenous	Ref					
Gestational weeks at enrolment						
<23 weeks	Ref					
≥ 23 weeks	0.36	-2.60, 3.33	0.809	0.71	-2.83, 4.26	0.688
Postcodes IRSAD [a] classification						
Disadvantaged area	Ref					
Advantaged area	0.54	-2.42, 3.50	0.719	-0.12	-3.77, 3.53	0.947
Pre-pregnancy BMI						
BMI (<25 kg/height in m^2)	Ref					
BMI (≥25 kg/height in m^2)	1.93	-1.75, 5.61	0.299	0.33	-3.33, 3.98	0.861

[a] IRSAD - Index of Relative Socio-economic Advantage and Disadvantage

[b] Adjusted for age, Aboriginal status of mother, gestational weeks at enrolment, BMI and IRSAD classification

Supplementary Table S4. Sensitivity analysis of linear regression of ARFS total score at enrolment by maternal characteristics including COVID-19 in the original model

	**Unadjusted β-Coefficient**	**95% CI**	**p value**	**Adjusted [b] β-Coefficient**	**95% CI**	**p value**
Age group						
≤25 years	Ref					
>25 years	3.22	0.42, 6.02	0.024	3.42	0.55, 6.29	0.02
Aboriginal status of mother						
Aboriginal and/or Torres Strait Islander	-1.49	-5.11, 2.13	0.417	-0.39	-3.97, 3.20	0.83
Non-Indigenous	Ref					
Gestational weeks at enrolment						
<23 weeks	Ref					
≥ 23 weeks	0.36	-2.60, 3.33	0.809	1.93	-1.13, 5.00	0.215
Postcodes IRSAD [a] classification						
Disadvantaged area	Ref					
Advantaged area	0.54	-2.42, 3.50	0.719	0.30	-2.66, 3.25	0.842
COVID-19 years						
Pre-COVID	Ref					
During COVID	-2.41	-5.33, 0.51	0.106	-3.31	-6.48, -0.15	0.04

[a] IRSAD - Index of Relative Socio-economic Advantage and Disadvantage

[b] Adjusted for age, Aboriginal status of mother, gestational weeks at enrolment, COVID-19 and IRSAD classification

Supplementary Table S5 Paired comparison of 24-hour food serves between early and late pregnancy visits

	**First pregnancy visit Median 23 weeks**		**Late pregnancy visit Median 33 weeks**		**McNemar test** ***p value***
Food group servings			Not meeting recommendations	Meeting recommendations	
	n	%	*n*	*n*	
Vegetables (servings/day)					
Not meeting recommendations	121	91.7	114	7	0.629
Meeting recommendations	11	8.3	10	1	
Fruits (servings/day)					
Not meeting recommendations	97	72.9	62	35	0.004
Meeting recommendations	36	27.1	14	22	
Soft drink [c] (servings/day)			≥ 1 serve on the previous day	less than 1 serve on the previous day	
≥ 1 serve on the previous day	75	55.2	60	15	0.088
less than 1 serve on the previous day	61	44.8	27	34	
Snacks [a c] (servings/day)					
≥ 1 serve on the previous day	90	66.7	63	27	1.00
less than 1 serve on the previous day	45	33.3	28	17	

[a] snack food (e.g. hot chips, potato chips, biscuits, donuts, cakes)

[b] According to the ADG food group recommendations for pregnant women.

[c] ADG recommends only occasional and in small amounts for energy-dense, nutrient-poor foods. Soft drink or snack foods - Representing women who had no or less than 1 serve of soft drink, cordial or juice, or less than 1 serve of snack foods.

Supplementary Table S6. Total gestational weight gain by pre-pregnancy BMI category

**BMI category**	**Total weight gain category at last observation**	***n* (%)**
Underweight (*n*=5)	Inadequate	1 (20.0)
	Adequate	1 (20.0)
	Excessive	3 (60.0)
Healthy weight (*n*=20)	Inadequate	8 (40.0)
	Adequate	3 (15.0)
	Excessive	9 (45.0)
Overweight (*n*=8)	Inadequate	2 (25.0)
	Adequate	0
	Excessive	6 (75.0)
Obesity (*n*=19)	Inadequate	3 (15.8)
	Adequate	4 (21.0)
	Excessive	12 (63.2)

Supplementary Table S7. Sensitivity analysis of gestational weight gain by each engagement component

	**Engagement initiation**		*p value*	**Engagement duration**		*p value*	**Engagement intensity**		*p value*
	**>23 weeks**	**≤23 weeks**		**≤12 weeks**	**>12 weeks**		**<2 bundles**	**≥2 bundles**	
Total weight gain category									
Adequate	1 (4.2)	7 (24.1)	<0.001	2 (6.7)	6 (26.1)	0.001	5 (13.5)	3 (20.0)	0.435
Inadequate	2 (8.3)	12 (41.4)		4 (13.3)	10 (43.5)		8 (21.6)	5 (30.3)	
Excessive	21 (87.5)	10 (34.5)		24 (80.0)	7 (30.4)		24 (64.9)	7 (46.7)	
Incremental weight gain category									
All pregnant women (*n*=52) [Mean (SD) kg/week]	0.52 (0.35)	0.37 (0.28)	0.091	0.49 (0.34)	0.37 (0.29)	0.197	0.46 (0.32)	0.41 (0.31)	0.649
Women with positive weight change [Mean (SD) kg/week] (*n*=46)	0.61 (0.26)	0.44 (0.22)	0.019	0.56 (0.28)	0.46 (0.22)	0.186	0.54 (0.25)	0.46 (0.27)	0.396
